# First lift-off and flight performance of a tailless flapping-wing aerial robot in high-altitude environments

**DOI:** 10.1038/s41598-023-36174-5

**Published:** 2023-06-02

**Authors:** Shu Tsuchiya, Hikaru Aono, Keisuke Asai, Taku Nonomura, Yuta Ozawa, Masayuki Anyoji, Noriyasu Ando, Chang-kwon Kang, Jeremy Pohly

**Affiliations:** 1 Graduate School of Science and Technology, Shinshu University, Nagano, 3868567 Japan; 2 Department of Mechanical Engineering and Robotics, Shinshu University, Nagano, 3868567 Japan; 3 Graduate School of Engineering, Tohoku University, Miyagi, 9808579 Japan; 4 Institute of Fluid Science, Tohoku University, Miyagi, 9808577 Japan; 5 Interdisciplinary Graduate School of Engineering Sciences, Kyushu University, Fukuoka, 8168580 Japan; 6 Department of System Life Engineering, Maebashi Institute of Technology, Gunma, 3710816 Japan; 7 Department of Mechanical and Aerospace Engineering, University of Alabama in Huntsville, Huntsville, AL 35899 USA

**Keywords:** Mechanical engineering, Aerospace engineering

## Abstract

Flapping flight of animals has captured the interest of researchers due to their impressive flight capabilities across diverse environments including mountains, oceans, forests, and urban areas. Despite the significant progress made in understanding flapping flight, high-altitude flight as showcased by many migrating animals remains underexplored. At high-altitudes, air density is low, and it is challenging to produce lift. Here we demonstrate a first lift-off of a flapping wing robot in a low-density environment through wing size and motion scaling. Force measurements showed that the lift remained high at 0.14 N despite a 66% reduction of air density from the sea-level condition. The flapping amplitude increased from 148 to 233 degrees, while the pitch amplitude remained nearly constant at 38.2 degrees. The combined effect is that the flapping-wing robot benefited from the angle of attack that is characteristic of flying animals. Our results suggest that it is not a simple increase in the flapping frequency, but a coordinated increase in the wing size and reduction in flapping frequency enables the flight in lower density condition. The key mechanism is to preserve the passive rotations due to wing deformation, confirmed by a bioinspired scaling relationship. Our results highlight the feasibility of flight under a low-density, high-altitude environment due to leveraging unsteady aerodynamic mechanisms unique to flapping wings. We anticipate our experimental demonstration to be a starting point for more sophisticated flapping wing models and robots for autonomous multi-altitude sensing. Furthermore, it is a preliminary step towards flapping wing flight in the ultra-low density Martian atmosphere.

## Introduction

Aerodynamic forces generated during flight such as lift and drag are proportional to the air density, squared of the wing speed, wing area, and respective force coefficient. Accordingly, as the density reduces, there must be a commensurate increase in the product of the remaining terms such that the lift can offset the weight of the vehicle to stay aloft. This is a critical consideration as the atmospheric density is a function of temperature and pressure which change with the flight altitude of an aircraft. For example, the air density on top of the Mt. Everest (approximately 9000 m) is about one third the density at sea level. Thus, for an aircraft to generate the lift to balance its weight at this high altitude, it must increase the product of wing area, reference velocity squared, and lift coefficient by at least three times what is required at the sea level.

Additionally, fluid dynamic flows are characterized by the Reynolds number (Re) which reduces with decreased atmospheric density for a given wing size. In these low Re environments, aerodynamic forces generated by fixed wings and rotating rotor blades are most likely degraded due to the flow separation and vortex shedding^1,2^. On the other hand, biological flyers such as insects and birds typically operate in a low Re regime (*O* (10^2^)–*O* (10^4^))^3–5^. They can generate large aerodynamic forces by effectively utilizing the unsteady aerodynamic mechanisms^5,6^, which prevail in this low Re flow regime. These unsteady mechanisms include the delayed stall of the leading-edge vortex^7^, the rotational circulation^8^, the wake capture^8^, and the clap-and-fling^9^, generated by flapping their thin flexible wings^5,10^. As such, bumblebees can fly on Mt. Everest by flapping their wings widely to account for the low-density conditions^11^. Monarch butterflies can effectively fly in thinner air by producing high lift coefficients by adjusting its body attitude and flight speed^12^.

An extremely low-density condition appears in flights on Mars which is one hundredth the air density of Earth. Recent success with National Aeronautics and Space Administration (NASA)'s Mars helicopter ‘Ingenuity’ demonstrated the feasibility of aerial exploration in the Martian environment^13^. However, generation of sufficient aerodynamic forces and control torques under the low-density conditions for long periods of time with meaningful payload capacity remains challenging in high altitude terrestrial and Martian environments. Recent theoretical and computational studies suggest the feasibility of highly efficient flapping wing robots on Mars^14–18^, including a bioinspired scaling relationship^14,15^. Nevertheless, flight experiments have not been performed.

In the past decade, various types of bioinspired flapping wing aerial robots have been developed to mimic the superior flight performance of biological flyers^19,20^. The Nano-hummingbird as the first tailless two-wing flapping robot with the ability to sustain hover and fly forward^21^. This was succeeded by the DelFly Nimble which has two wings on each side of the robot and can mimic the rapid escape maneuvers of flies^22^. The robotic hummingbird can perform goal-directed maneuvers, rapid turns, and 360° body flips by implementing reinforced learning^23–25^. Additionally, the KU-beetle that is inspired by a species of horned beetles and achieved 8.8 min of flight^26^. Thus, it has been demonstrated that flapping-wing aerial robots are capable of agile, bio-inspired flight. Their maneuverability, small size, and lightweight designs, and their low Re characteristics make them promising candidates for use in various environments such as disaster sites, indoor security, and aerial exploration at high altitudes on Earth and on Mars where traditional fixed and rotary wings may not be as capable.

Very few researchers have conducted experiments for flapping-wing aerial robots under low-density environments like those at high altitudes on the earth. One of the first experiments was in a vacuum chamber, where the effects of inertial forces on hummingbird-like wings were quantified^27^. However, no lift was generated as there was no surrounding fluid. More recently, a bird-like flapping-wing aerial robot with large wings achieved forward flight in a high-altitude environment of 4500 m^28^ where the density was about half that of sea-level. The results showed that increasing flight speed and frequency was required to compensate for the loss of lift and drag due to reduced air density. In the ultra-low Martian density conditions, X-wing flapping wings showcased the possibility of sufficient lift generation under very low-density conditions^29,30^. However, the flapping amplitude was much lower than those of flying animals and the experimental data remain limited. One common challenge identified in these studies is that the nature of the wing deformations of thin flapping wings in thinner air is inherently different than at the sea-level. The resulting wing motion is a priori unknown because it is in dynamic balance with the surrounding aerodynamics. As such, it is difficult to design and develop flapping wings and motions that produce sufficient lift to sustain a target vehicle weight. Another reason is that it is difficult to conduct experiments in low density conditions compared to in air (sea-level) or in water. Further, it is difficult to observe the motion of flying animals in thinner air. As such, we cannot simply mimic the animal morphology and motion. We need a better understanding of the underlying physical principles to scale the size and motion.

Here we report on the feasibility of high-altitude flight by a developing a tailless flapping-wing aerial robot. The objective of this study is two-fold: First, we measure the wing motion and generated aerodynamic forces in an air density range of 0.360 kg/m^3^ (9000 m) and 1.184 kg/m^3^ (sea level) for scaled wing size and motion frequency and compare to a bioinspired scaling relationship for flexible flapping wings^31,32^. Second, we test if the considered wing size and motion produces a lift-off of the robot in the lowest air density of 0.360 kg/m^3^. This study demonstrates the feasibility of bioinspired flight in high-altitude conditions by a coordinated scaling of the wing size and motion frequency. The optimization of the wing design in terms of flapping frequency, wing shape/aspect ratio and wing flexibility, and vehicle size was not conducted and left for future work.

### Tailless flapping-wing aerial robot, wing kinematics, and objective conditions

**Figure 1:**
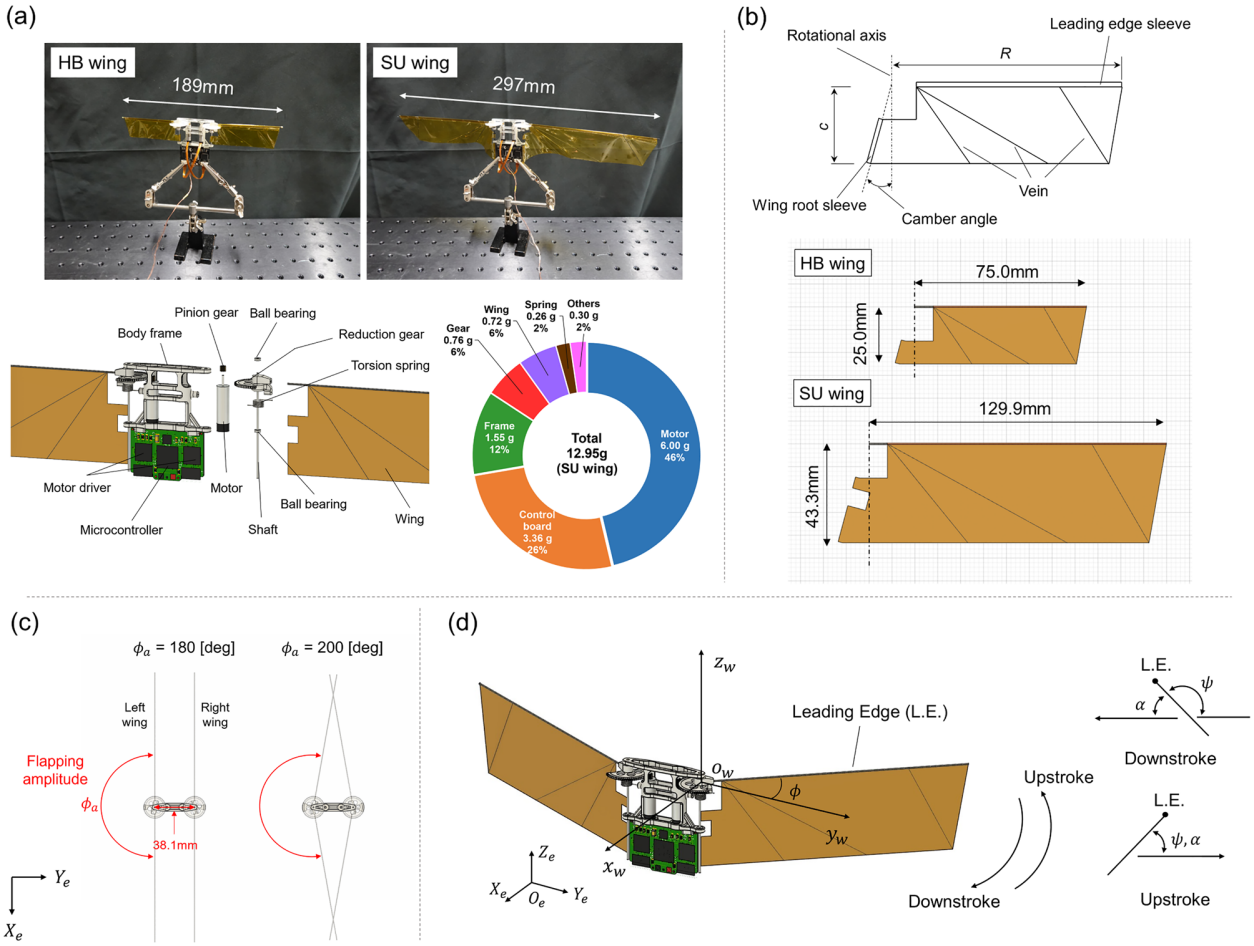
(**a**) Pictures and computer-aided design image of robo-hummingbird Shinshu and weight breakdown of the robo-hummingbird Shinshu with SU wing (larger wing considered in this study). (**b**) Design of the wing. Two types of wing shape used in the lift measurement. *R* and *c* indicate the single wing length and chord length. (**c**) Schematics of the distance between left and right wing of the robo-hummingbird Shinshu with SU wing and influence of the flapping amplitude (*ϕ*_*a*_) on the distance between two wings. The wings touch each other when *ϕ*_*a*_ approaches more than 196 degrees. (**d**) Definition of coordinates and angles of the wing motion. L.E., *α*, *ϕ*, and *ψ* denote the leading edge of the wing, the angle of attack, the flapping angle, and the wing rotational angle, respectively.

Figure 1a shows the design of the robo-hummingbird Shinshu, a tailless flapping-wing aerial robot developed in this study. It is inspired by the hummingbird-like flapping-wing robot developed by Tu et al.^23^. The driving system of the robot is a direct type actuation that consists of brushless motors (ECXSP06M, Maxon), reduction gears, torsional springs, and two wings. The motor accounts for almost half of the robot’s weight (Fig. 1a). The direct type wing actuation system was inspired from those of hummingbirds^33,34^ and the hummingbird-like flapping wing robot^23^. The torsional spring (33-0437 for the right wing, 34-0437 for the left wing, Samini Co., Ltd) can restore energy and help generate smooth flapping motion. The body frame is made of acrylic resin using a 3D printer (AGILISTA-3200, KEYENCE Corp.). The flapping motion is reproduced by repeating the forward and reverse rotation of the motor. The motion is controlled by a microcontroller and a motor driver that are mounted on the control board. The driving power of the robot is supplied from an external power source (SPPS-C-3010B, IKococater) through a cable.

The wing is made up of a 12.5 μm polyimide film and 0.3 mm carbon rods as veins. The leading edge (L.E.) is made of 1.0–0.5 mm carbon pipe. Figure 1b shows the schematics of the wing planform. The camber part at wing root and sleeve part attached to the L.E. are capable of a passive wing rotation and twist. The camber angle is set to 15 degrees motivated by previous studies^35,36^. Two types of wings are used: Wing1 mimics the wing size of hummingbirds^33,34^ and the hummingbird-like flapping-robot^23^, designed for the standard sea-level conditions named HB wing; Wing2 has a wing area three times that of HB wing named SU wing, respectively, while maintaining a constant aspect ratio (Fig. 1b). The total mass of robot is 12.39 g and 12.95 g when the HB wing and SU wing are attached, respectively. Note that the wing-to-body mass ratio is 1.93% and 5.56% that are within the range of biological flyers^37–39^. The distance between left- and right-wing roots is 1.2*c* where *c* is the chord length. As such the wings are allowed to rotate more than 180 degrees. If the flapping amplitude (*ϕ*_*a*_) becomes more than 196 degrees, and then, the wings of the robo-hummingbird Shinshu with SU wing touch each other, allowing for clap-and-fling motion to occur^5,6,9^ (Fig. 1c). The flapping frequencies (*f*) of the HB and SU wings are 22.7 Hz and 10.9 Hz, respectively. The same amount of electric power was provided to the wing motors in all experiments.

Figure 1d shows the coordinate systems and definition of flapping parameters. $O_{e} X_{e} Y_{e} Z_{e}$ is the coordinate system on the earth, while $o_{w} x_{w} y_{w} z_{w}$ is the coordinate system attached to the intersection point of wing rotational axis and L.E.. The flapping angle *ϕ* is defined as the angle between the *y*-axis and the L.E. of the wing; the angle of attack α is defined as the angle between the flow relative to the wing motion and the wing chord and is closely related to the wing rotational angle^40^ *ψ*; the *ψ* is the angle between the stroke plane and the wing chord; and the flapping amplitude *ϕ*_*a*_ is defined as *ϕ*_*max*_–*ϕ*_*min*_. Only the flapping motion is actuated. The wing rotational angle is a non-actuated passive motion due to the wing flexibility.

**Figure 2:**
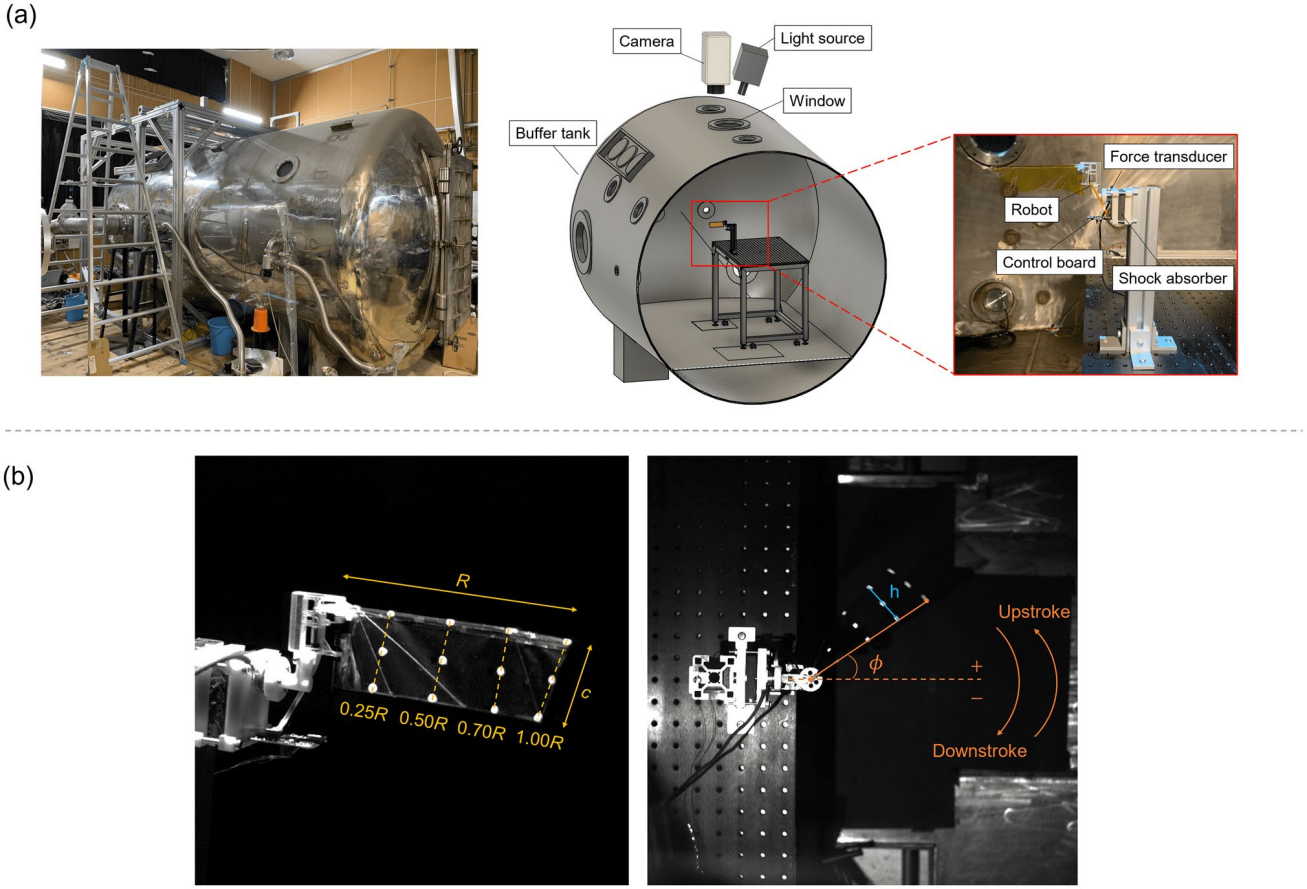
(**a**) Overview of buffer tank and system of simultaneous measurement. (**b**) Position of the markers on the wing (left) and top view of the wing shape measurement system (right). *R*, *c*, *ϕ*, and *h* denote the single wing length, the chord length, the flapping angle, and the horizontal distance between the two extreme marker points on the wing.

To simulate the high-altitude environment, the air density in the buffer tank attached to the low-density wind tunnel^41,42^ located at the Institute of Fluid Science, Tohoku University in Japan was controlled by changing the pressure inside the tank per measured temperature (Fig. 2). In this study, five flight altitudes were considered: 1.184 kg/m^3^, 0.921 kg/m^3^, 0.658 kg/m^3^, 0.592 kg/m^3^, and 0.360 kg/m^3^ correspond to the flight altitudes of sea level, 2243 m, 5427 m, 6477 m, and 9000 m, respectively.

The main parameters are shown in Table 1. The reference velocity $U_{{{\text{ref}}}} = 2\pi f\phi_{a} R_{2}$ depends on the radius of second moment of area^43^, $R_{2} = \sqrt {1/S\mathop \smallint \limits_{0}^{R} cr^{2} dr}$, where *c* is the chord length at any radial position *r* and *R* is the wing length. For the considered wing shapes, *R*_2_/*R* = 0.56 is a constant.

**Table 1:** The dimensionless and dimensional parameters of wing design and kinematic^5,37,38,48^.

	Parameters	Symbol	Robot (HB wing) on 1.184 kg/m^3^	Robot (SU wing) on 0.360 kg/m^3^	Bumblebee^37,48^	Hummingbird^38^	Insect range^5,37,38^
Dimensionless	Wing aspect ratio	*AR* = *R*/*c*	3.0	3.0	3.3	4.1	${2}{ \leqq }AR{ \leqq }5$
	Reduced frequency	$k = \pi fc/U_{tip}$	0.15	0.13	0.24	0.15	$0.1{ \leqq }k{ \leqq }0.4$
	Reynolds number	$Re = \rho_{a} U_{tip} c/\mu_{a}$	18,786	9767	2140	13,891	*O* (10^2^–10^4^)
	Wing tip Mach number	$M_{tip} = U_{tip} /a$	0.034	0.034	0.024	0.030	${\text{M}}_{{{\text{tip}}}} { \leqq } 0.1$
	Mean lift coefficient	$C_{L} = F_{a} /\left( {0.5\rho_{a} U_{tip}^{2} S} \right)$	0.57	0.51	0.80	0.73	*O* (10^−1^–10^0^)
	Wing-to-body mass ratio	$m^{*} = m_{w} /m$	1.93%	5.56%	0.47–0.74%	5.1–7.0%	0.1% < m* < 15%
Dimensional	Mass (g)	*m*	12.39	12.95	0.18	8.4	
	Flapping frequency (Hz)	*f*	22.7	10.9	155.0	23.3	
	Wing length (mm)	*R*	75.0	129.9	13.2	85.0	
	Surface area of single wing (mm^2^)	*S*	1875	5625	52.9	1753	
	Flapping amplitude peak-to-peak (deg)	*ϕ*_*a*_	197	233	116	151	
	Reference velocity (m/s)	*U*_*r*ef_	6.56	6.56	-	-	
	Average wing tip velocity (m/s)	*U*_*tip*_	11.71	11.71	8.28	10.44	

Note that the reduced frequency, the Reynolds number, and the mean lift coefficient were calculated based on the average wing tip velocity ($U_{tip} = 2\phi_{a} fR$) and the wing chord length to enable comparison to insect data in the literature. Here, *ρ*_*a*_ is the air density corresponding the altitude, *μ*_*a*_ is the viscosity coefficient corresponding the altitude, *F*_*a*_ is the mean lift force of the robot and the weight of insects, the gravitational acceleration is 9.81 m/s^2^, *S* is the single wing area, and *m*_*w*_ is total mass wing mass (the mass of wing pair). The Strouhal number becomes a constant for hover and is $St = \phi_{a} ARk/\pi = R/\left( {2\pi R_{2} } \right) = 0.284$ for the robo-hummingbird Shinshu.

High-speed cameras were used for measuring the flapping amplitude and wing rotational angles at frame rates of 960 Hz and 2000 Hz. The lift generated by the wing was simultaneously measured using a force transducer. Further details can be in the Methods section.

## Results

**Figure 3:**
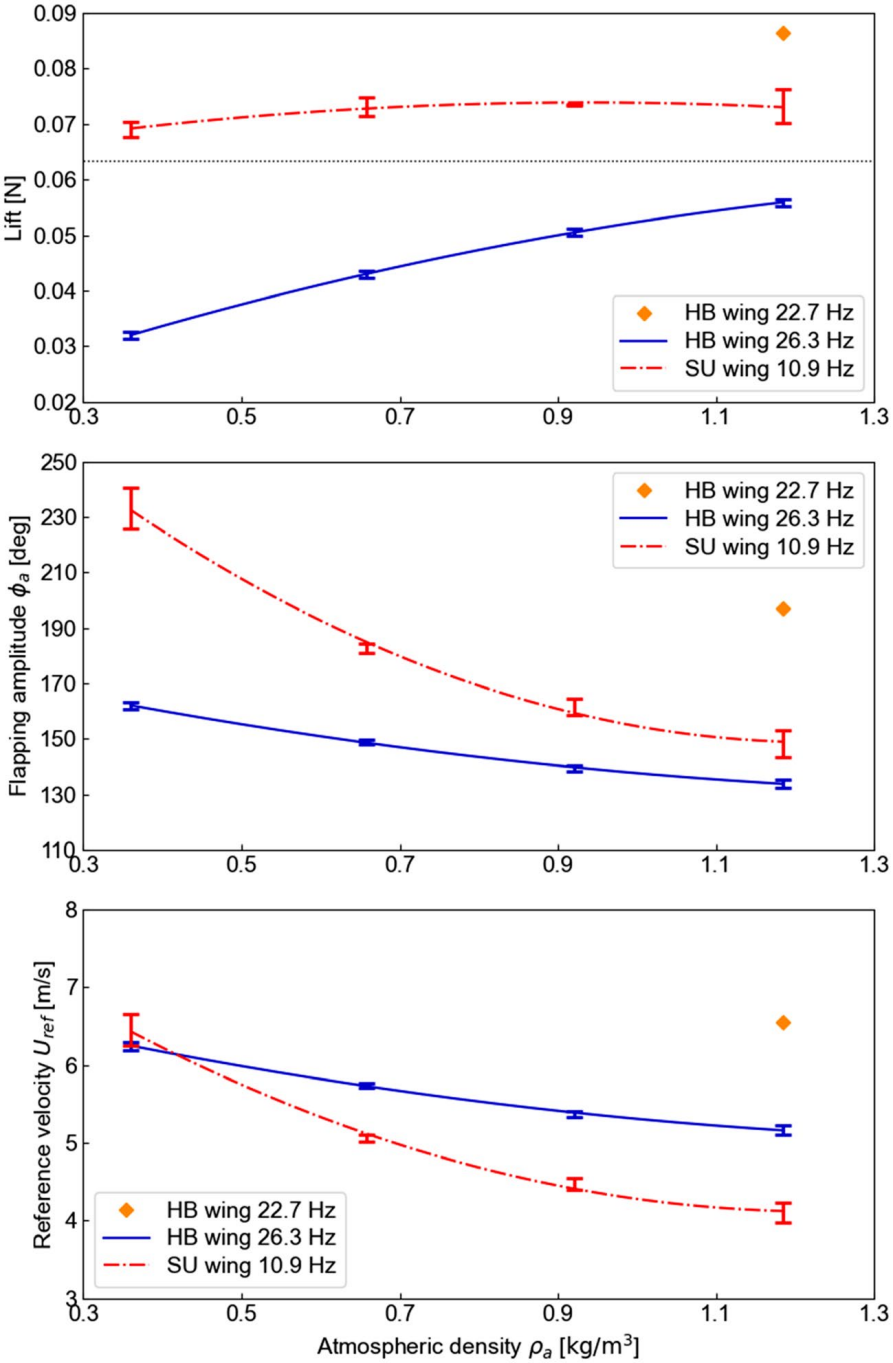
Results of simultaneous measurement of lift (top) and flapping amplitude *ϕ*_*a*_ of the wings (middle) and a reference velocity *U*_*ref*_ (bottom). The reference velocity is the average wing velocity at the spanwise location of the center of the second moment of area of the wing (about flapping axis). Note that each wing flaps with a different frequency. The flapping wing frequency of each wing is determined by the flapping amplitude at the air density at the altitude of 0 m. The black dotted line in the top plot is half of the robot weight, the target value for lift. Diamond symbol indicates the result corresponding to the normal operation under the altitude of 0 m (sea level).

We measured how the decrease in the atmospheric density affected the aerodynamic force generation and wing motion (Fig. 3). The HB wing generated a lift of 0.17 N, which exceeds the vehicle weight of 0.12 N at a flapping frequency of 22.7 Hz at the sea-level condition. We employed two approaches: i) Use the HB wing at a higher frequency of 26.3 Hz; ii) Use a scaled-up SU wing at a lower frequency of 10.9 Hz. The faster flapping HB wing was unable to generate sufficient lift, which reduced with the altitude. For the SU wing, the lift only reduced by 6% in the lowest density environment compared to the sea-level conditions despite a reduction of air density by 33%. The SU wing was able to generate enough lift to support its own weight of 0.13 N under all altitude conditions considered. The lift peaked when *ψ* was at the extrema.

**Figure 4:**
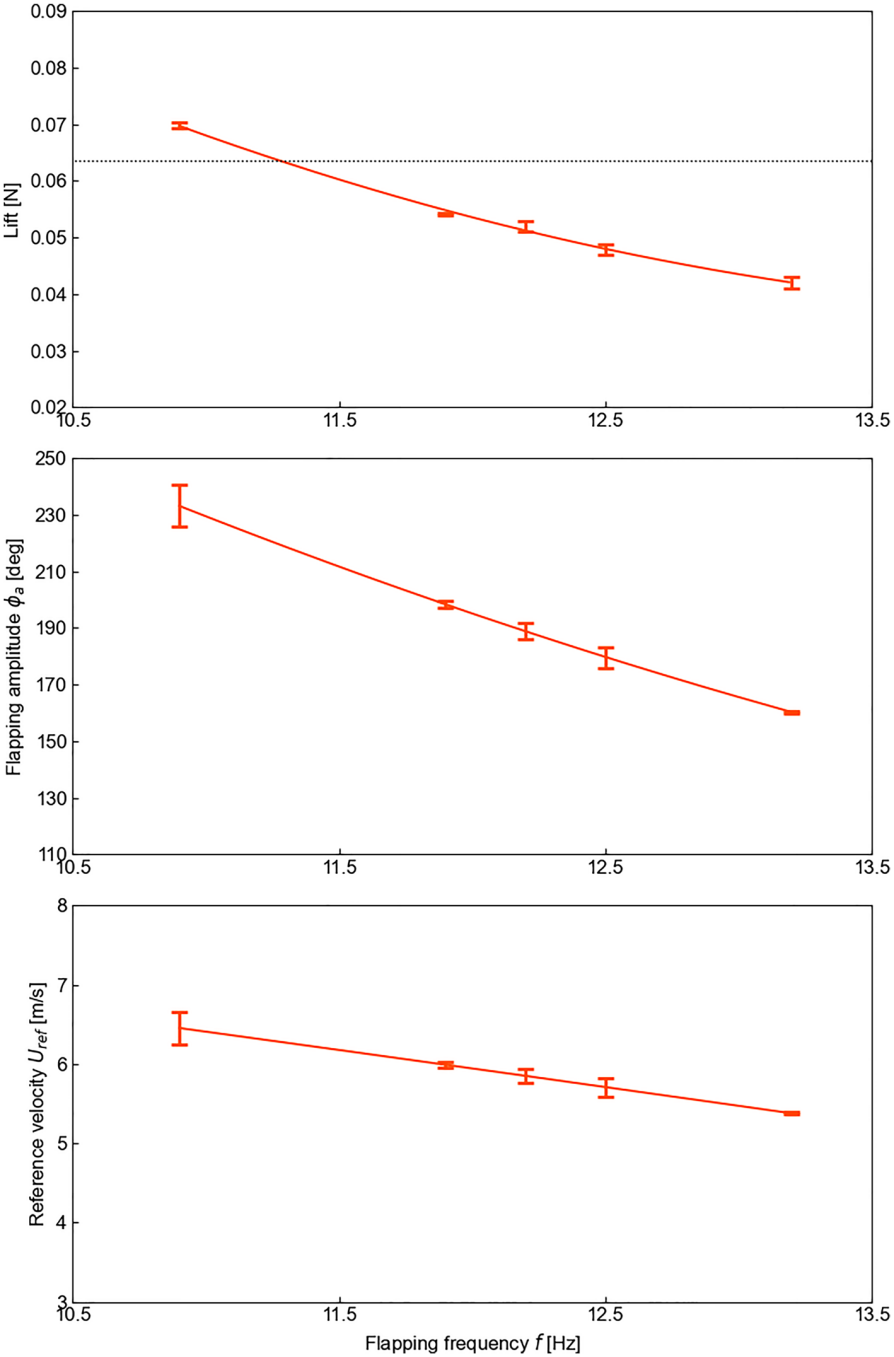
Results of simultaneous measurement of the SU wing: Lift (top) and flapping amplitude *ϕ*_*a*_ of the wing (middle) and a reference velocity *U*_*ref*_ (bottom) as a function of the flapping frequency at high-altitude condition (9000 m). The reference velocity is the average wing velocity at the spanwise location of the center of the second moment of area of the wing (about flapping axis). The black dotted line in the top plot is half of the robot weight, the target value for lift.

**Figure 5:**
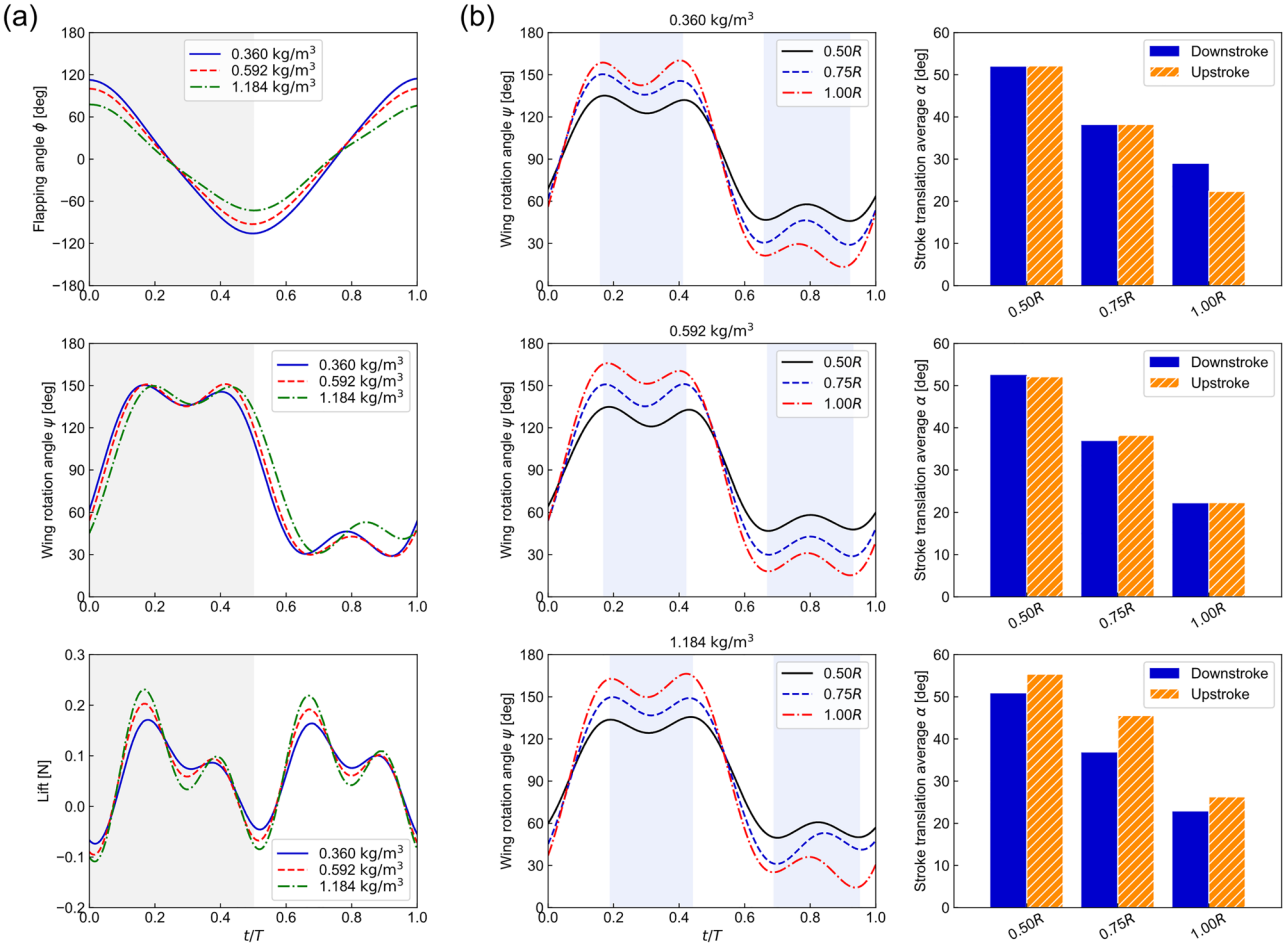
(**a**) Result of measurement of flapping angle *ϕ*, wing rotation angle *ψ* and lift (the SU wing, 10.9 Hz) of 0.75*R* at each density condition. (**b**) Result of wing rotation angle *ψ* at the three single wingspan locations (0.50*R*, 0.75*R*, and 1.00*R*) of SU wing and average *α* during stroke translation at each density condition. Blue colored regions denote stroke translation of the half stroke.

There are three factors contributing to the lift generation of the SU wing. The first factor is the increased wing size. As the lift is proportional to the wing area, the use of a larger wing improved the lift generation. The second factor is the increased wing speed under the reduced density condition. The wing speed is measured at *R*_2_, such that $U_{{{\text{ref}}}} = 2\phi_{a} fR_{2}$.The flapping amplitude *ϕ*_*a*_ increased as the atmospheric density was decreased (Fig. 3). The increase in *ϕ*_*a*_ was more pronounced particularly in the low-density conditions for the larger wing (the SU wing). The increase in *ϕ*_*a*_ is purely due to the wing deformations of the larger thin wings, which was affected by both the inertial and aerodynamic forces, which in turn depends on the density. As the flapping frequency was held constant for each wing, the wing speed increased, which has a quadratic growth rate on the lift. Counterintuitively, the lift reduced when the flapping frequency increased (Fig. 4). The increase of the flapping frequency leads to a lower *ϕ*_*a*_ and consequently a lower lift. The third factor is the passive pitch motion. Due to the wing flexibility, the wing passively rotates to achieve its pitch motion. The wing rotation amplitude at 75% of the wing length *R* (0.75*R*) remained almost constant despite changing the air density (Fig. 5). The averaged angle of attack at 0.75*R* during the translational motion in the stroke under the high-altitude condition was 38.2° (minimum of 29.0° and maximum of 46.5°), which is within the same range as insects, thus leading to bio-inspired lift generation. The timing of the wing rotation was slightly earlier as the air density decreased due to the decrease in aerodynamic damping. The passive wing rotational dynamics are primarily due to the interaction between aerodynamic forces that lag the wing motion and the wing inertia forces^5,27^ which are in phase with the motion. The inertial forces have more impact on the passive wing rotation under the low-density condition since the wing mass increases while the aerodynamic damping reduces.

**Figure 6:**
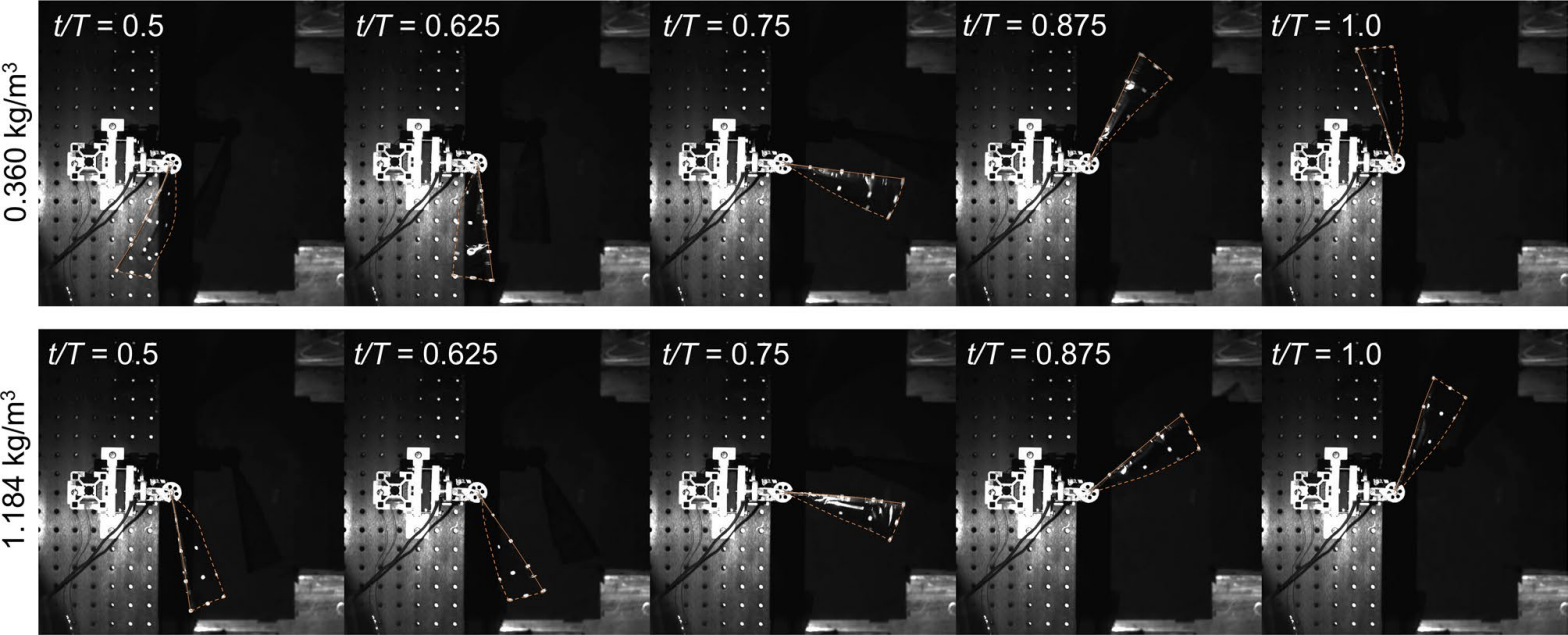
Snapshots of the shape of SU wing during the upstroke of a flapping cycle at 0.360 kg/m^3^ (top) and 1.184 kg/m^3^ (bottom) of the air density corresponding to the altitude of 9000 m and 0 m (sea level). The outline of the wing is highlighted with an orange line and solid line indicates the leading edge of wing. *t* and *T* denote the time instant of a flapping wing and the period of a flapping cycle.

One potential mechanism for the reduction of the aerodynamic damping is the wing twist. The *α* was the largest at the wing root and reduced towards the tip (Fig. 5). The lower *α* near the tip, where the velocity is the highest, may be related to a smaller induced drag^44^. Figure 6 shows the instantaneous shape of SU wing during the flapping motion under sea-level and high-altitude conditions. Although there was a difference in the *ϕ*_*a*_, the passive wing rotation and the overall three-dimensional wing shapes remained similar.

**Figure 7:**
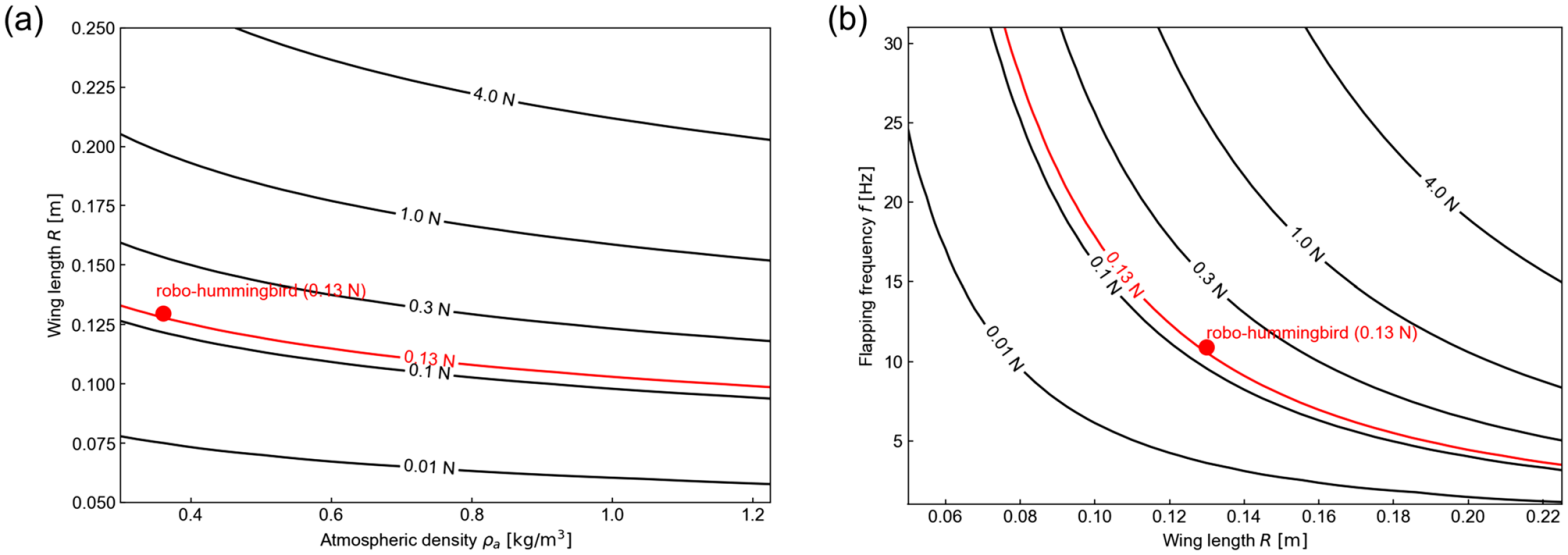
Qualitative trends predicted by the *γ*-scaling model^30,31^. Red circles represent the robo-hummingbird Shinshu parameters that result in sufficient lift to offset its weight in hover (0.13 N). (**a**) The wing length *R* as a function of the air density *ρ*_*a*_ for various vehicle weights at a flapping frequency of 10.5 Hz, an aspect ratio of 3, and a flapping amplitude of 89.5 degrees. (**b**) The flapping frequency *f* as a function of the wing length *R* at the lowest air density condition of 0.360 kg/m^3^ at an aspect ratio of 3 and a flapping amplitude of 89.5 degrees.

The lift generation of flexible flapping wings depends on the wing shape deformation and motion, which, in turn, hinges on the lift on the wing. To analyze the qualitative trends between the wing length, flapping frequency, and air density, the *γ*-scaling^30^ was used. The scaling parameter $\gamma = \left( {1 + 4\rho^{*} h_{s}^{*} /\pi } \right)St k/\left[ {{\Pi }_{0} \left\{ {\left( {f_{1} /f} \right)^{2} - 1} \right\}} \right]$ is the relative shape deformation parameter, which scales with the wing passive wing rotations. Definitions of the employed nondimensional parameters pertaining to the fluid–structure interaction and the values corresponding to the robo-hummingbird Shinshu are shown in Table 2. This scaling method was validated through insect data^30^ and computational and experimental flexible flapping wing data for air and water^30,31^. The lift coefficient on a flexible flapping wing in hover scales as $C_{L} = {\Pi }_{1} St c_{\gamma } \gamma^{1.19} /\left( {\rho^{*} h_{s}^{*} k} \right)$, where $c_{\gamma } = 10^{0.98}$. Since the lift is in equilibrium with the weight in hover, the weight can be expressed as $W = L = n_{{{\text{wings}}}} \rho_{f} U_{{{\text{ref}}}}^{2} SC_{L} /2$, where there are two wings *n*_wings_ = 2. Figure 7 shows the qualitative trends between the wing length *R*, the flapping frequency *f*, and the air density *ρ*_*a*_ as a function of the vehicle size expressed in weight. In general, larger wings and slower motions are required as the density reduces. In particular, the robo-hummingbird Shinshu with SU wing length of 0.1299 m and frequency of 10.9 Hz closely agree with the scaling analysis at the lowest density condition of 0.360 kg/m^3^ for the weight of 0.13 N of the robo-hummingbird Shinshu with SU wing.

**Table 2:** Nondimensional parameters pertaining to the flexible flapping wing γ-scaling^30^.

Parameters	Symbol	Robot (SU wing) at 0.360 kg/m^3^	Notes
Density ratio	$\rho^{*} = \rho_{s} /\rho_{a}$	4444	*ρ*_*s*_ = 1600 kg/m^3^ based on carbon fiber data
Wing thickness ratio	$h_{s}^{*} = h_{s} /c$	0.012	*h*_*s*_ = 1 mm based on the LE vein
First chordwise natural frequency	$f_{1} = k_{1}^{2} /\left( {2\pi } \right)\sqrt {Eh_{s}^{*2} /\left( {12\rho_{s} c} \right)}$	Variable	*E* = 181 GPa based on carbon fiber data
Effective inertia	${\Pi }_{0} = \rho^{*} h_{s}^{*} \left( {k/\pi } \right)^{2}$	Variable	
Effective stiffness	${\Pi }_{1} = Eh_{s}^{*,3} /\left( {12\rho_{a} U_{{{\text{ref}}}} } \right)^{2}$	Variable	
First beam vibrational eigenvalue	*k*_1_ = 1.875	1.875

**Figure 8:**
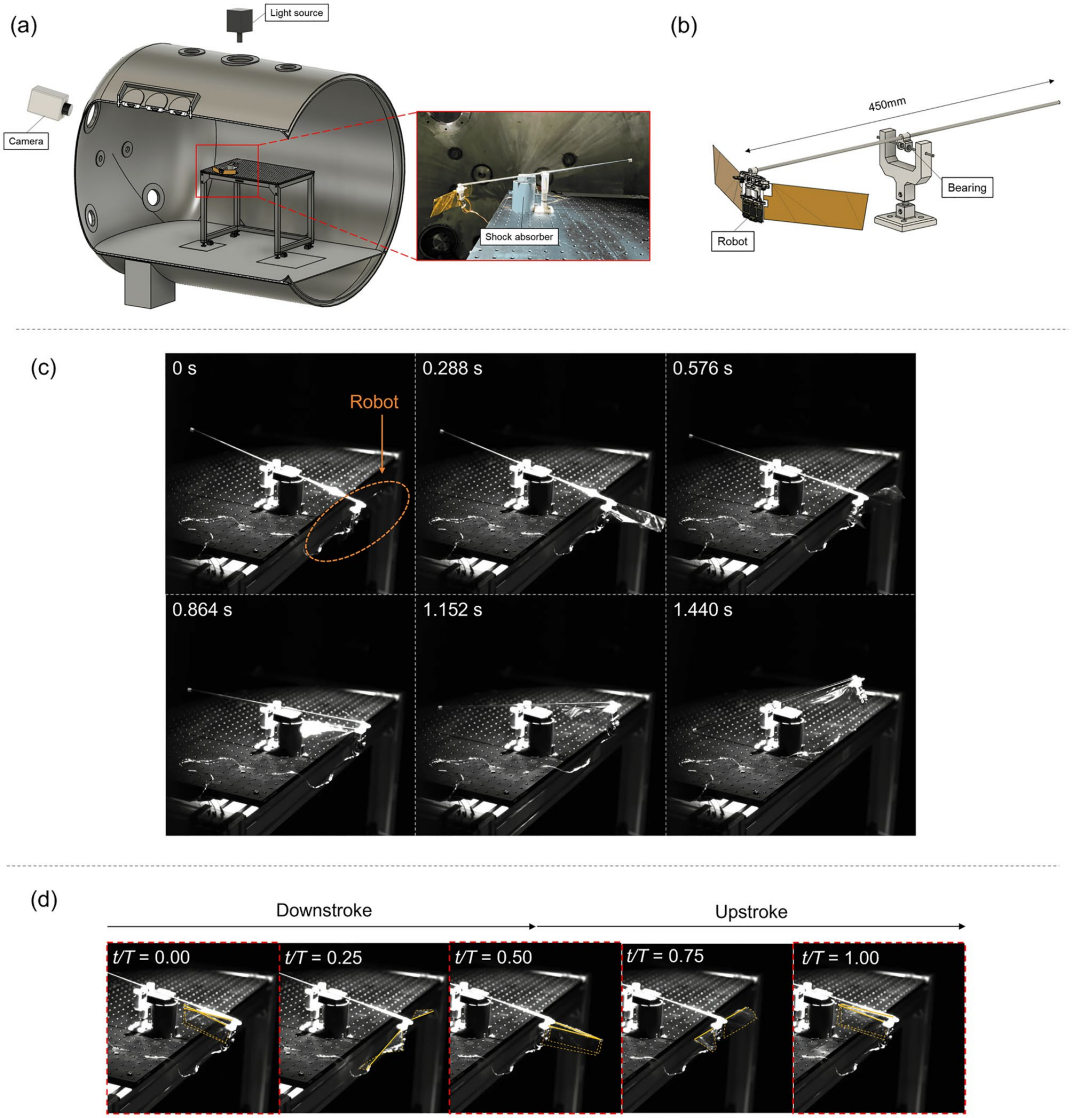
(**a**) System of flight demonstration test of the robo-hummingbird Shinshu with SU wing under the high-altitude condition (9000 m). (**b**) Design of balance type device. (**c**) Snapshot of flight demonstration (from 0 to 1.44 s). (**d**) Snapshots of flapping motion under the high-altitude condition. *T* denotes the period of flapping wing motion. The wings touched each other, suggesting that there could have been clap-and-fling motion at the start and the end of the stroke (*t*/*T* = 0.00, 0.50, and 1.00, respectively). The corresponding times are highlighted by the red dotted lines. Orange solid line indicates the L.E. of the wing.

Finally, motivated by the high lift measurement with SU wing under high-altitude condition and supported by the bioinspired scaling relationship analyses, we conducted a lift-off flight of the robo-hummingbird Shinshu with the SU wing. The experimental setup is shown in Fig. 8a,b. The air density was set to one third of the sea-level condition. Figure 8c showcases snapshots of the demonstration, documenting that robo-hummingbird Shinshu with SU wing successfully took off at high-altitude condition due to leveraging unsteady aerodynamic mechanisms unique to flapping wings (see Supplementary Videos S1 and S2). The robo-hummingbird Shinshu with SU wing flew upwards for a distance of 167.4 mm in 1.44 s. In addition, Fig. 8d shows that the wings of the robo-hummingbird Shinshu with SU wing touch each other, indicating a potential presence of clap-and-fling effects^5,6,9^ that could help increasing the lift generation.

## Discussion

To the best of our knowledge, this is the first experimental demonstration of the take-off of an insect-like tailless flapping-wing aerial robot in low-density, high-altitude condition (9000 m). Table 1 compares the main parameters related to the flight performance of the robo-hummingbird Shinshu under sea-level and high-altitude conditions to those of bumblebees, and hummingbirds under the sea-level condition. The resulting Mach numbers and the reduced frequencies suggest that the wings moved in the incompressible flow region and utilized the unsteady aerodynamic mechanisms^5,6^. The mean lift coefficient under high-altitude condition was slightly reduced compared to that under sea level. Each of the dimensionless parameters pertaining to the aerodynamics of robo-hummingbird Shinshu in the high-altitude condition overlapped the range of bumblebees and hummingbirds, which is an indication of a bioinspired scaling enabling a high lift. Further, the application of the *γ*-scaling suggests that the passive rotations due to wing deformation were preserved. The robo-hummingbird Shinshu is able to leverage the same unsteady aerodynamic mechanisms that insects and birds used to produce high lift.

Under the high-altitude condition (9000 m), the reduction in the air density, while keeping the flapping frequency the same, resulted in an increased flapping amplitude for the larger wing (the SU wing). This resulted in a wing speed in the high-altitude condition which as almost the same as that at the sea-level condition. Moreover, the variation in *α* during the flapping motions was minor for both flight-altitudes. By balancing and scaling these design parameters, the lift generated at the high-altitude condition remained similar to its sea-level counterpart. Similar mechanism was observed for insects in high-altitude enviroments^11^. This study aimed to provide experimental data of the forces and motions generated by flapping wings and a bioinspired scaling analysis in thinner air. The employed *γ*-scaling method captures the effects of fluid–structure interaction for various atmospheric densities. As such, this scaling analysis provides a qualitative trend that is consistent with the measured data: It is not a simple increase in the flapping frequency, but a coordinated increase in the wing size and reduction in flapping frequency enables the flight in lower density condition. It is anticipated that ultra-low-density environments enable flapping motion of the larger wings that would otherwise be too large to move under the sea-level condition. The key mechanism is to preserve the passive rotations due to wing deformation, confirmed by a bioinspired scaling relationship.

Now that the tailless flapping-wing aerial robot can generate the aerodynamic forces needed to fly at high-altitude, the next step is to achieve hover and forward flight using a robot with attitude control. In addition, the power was supplied from an external power source via cables in this study. A battery power would enable free flight and could realize a higher degree of freedom in flight. Furthermore, sensing and information gathering missions would require additional cameras, sensors, etc. To carry this payload a larger lift would be need through a more refined optimization study including wing shape and structure and flapping frequency.

Finally, it is envisioned that the resulting model could be extrapolated to the ultra-thin Martian atmosphere. Such a flapping robot could enable Martial aerial exploration assisting the rovers and human exploration^29,30^. The use of multiple flight vehicles could work together to observe weather and terrain data around the rover for route planning and guidance.

## Methods

### Measurement of aerodynamic force and wing motion

All experiments were conducted in a buffer tank attached to the low-density wind tunnel^41,42^ located at the Institute of Fluid Science, Tohoku University in Japan (Fig. 2a). The high-altitude conditions were reproduced by depressurizing the air inside of the tank and the low-density wind tunnel. The pressure inside the buffer tank and the low-density wing tunnel was depressurized by using an oil rotary vacuum pump (SOGEVAC SV 300B, Leybold) and a roots vacuum pump (RUVAC WAU 1001, Leybold) and measured using a digital pressure gauge (DG-920, Tokyo Aircraft Instrument). The temperature inside the tank was assumed to be same as the temperature inside the low-density wind tunnel that was measured using thermocouple attached to inside the low-density wind tunnel. Note that the method of estimation of air density in the tank can be found in the following section (i.e. Air density estimation). There is no mainstream. The diameter of the tank was 2.2 m and the length 4.3 m. The influence of interreference with the tank wall is sufficiently small for the main objective of the present study. Additional detailed information of the low-density wind tunnel can be found in Anyoji et al.^41^. The feasibility of conducting physical experiments of the rotating blade under low-density conditions was demonstrated in Nagata et al.^42^.

The experimental setup of the simultaneous measurement inside the tank is shown in Fig. 2a. For the simultaneous measurements, only one wing was used. The robot was attached to a force transducer (ATI Nano-17, ATI Industrial Automation) and fixed to a table inside the tank. The forces were recorded at a sampling rate of 2000 Hz. The output of the sensor was connected to a data acquisition board (USB-6341, National Instruments) and the saved to an external computer. The flapping amplitude *ϕ*_*a*_ was measured using either a high-speed camera (RX100VII, SONY) with the sampling frequency of 960 Hz or a high-speed camera (SA-X2, Photron) of the sampling frequency of 2000 Hz through a window at the top of the tank. The wing was illuminated by a light source to improve the quality of the image (PFBR-600, CCS Inc.). Considered densities ranged from the sea-level condition (1 atm; 1.184 kg/m^3^) to high-altitude conditions corresponding to an altitude of about 6500 m (1/2 atm, 0.592 kg/m^3^) and an altitude of about 9000 m (1/3 atm; 0.360 kg/m^3^). Both data were processed using a low pass filter with a cut-off frequency of 50 Hz. The driving frequency in each wing was set to be within the range of flapping amplitude from 130 to 160 degrees under the sea level. The flapping amplitude is a priori unknown due to the nonlinear fluid–structure interaction of the wing in preliminary tests. The mean values were calculated using three repeated trials. For the measurement of detailed wing motion, the wing motion was recorded using the high-speed camera and digitized by tracking the positions of markers placed on the wing. A photo of the wing with markers is shown in Fig. 2b. The markers were tracked using DLTdv8^45^ of MATLAB. The flapping angle *ϕ* was calculated by tracking the leading edge (L.E.). The angle of attack *α* is defined as

$$\alpha = \cos^{ - 1} \left( h/c \right),$$

where *h* is the horizontal distance between the two extreme marker points and *c* is the sectional chord length of the wing. The wing rotation angle *ψ*^31^ was calculated based on direction of the wing as:

$$\psi = \left\{ {\begin{array}{*{20}c} {\alpha \left( {in \,upstroke} \right)} \\ {\pi - \alpha \left( {in \,donwstroke} \right)} \\ \end{array} } \right.$$

It should be noted that the effective angle of attack plays an important role in flapping wing aerodynamics. In particular, the effective angle of attack depends on the ratio of the flapping wing speed and forward speed^46^ in addition to any body rotations^12^. However, in hover there is no forward velocity nor a wing motion component that is normal to this forward velocity. As such the main angle of attack is from the geometric angle of attack. Note that this geometric angle of attack *α* is not prescribed in the cases considered in this study. It is a combined outcome of the wing motion (inertia), the aerodynamic forces, and the structural effects.

### Air density estimation

The air density in the tank was estimated based on the following formula^47^.

$$\rho_{a} = \frac{{0.0034837P_{T} }}{T + 273.15},$$

where *T* and *P*_*T*_ are the temperature and pressure of the air in the tank, respectively. In this study, we measured *P*_*T*_ and assumed a constant *T* of 25 degrees of Celsius as the change in the tank temperature varied only by 1 to 2 degrees of Celsius.

### Lift-off flight demonstration test

The experimental setup is shown in Fig. 8a,b. The robot was mounted on a balance device with a joint. To offset the weight of the joint connecting the flapper to the balance, an additional joint was added on the opposite side, such that the flapper was lifting its own original weight of 0.27 g. The motion of body was limited to one degree of freedom, such that an attitude control was not required. Based on the results of the simultaneous measurement of the SU wing (Fig. 3), the robo-hummingbird Shinshu with SU wing was driven at 10.9 Hz.

## Supplementary Information

Supplementary Legends.

Supplementary Video 1.

Supplementary Video 2.

## Data Availability

The datasets used and/or analyzed during the current study available from the corresponding author on reasonable request.
