# Supplementary material for: First lift-off and flight performance of a tailless flapping-wing aerial robot in high-altitude environments
Source: Sci Rep. 2023 Jun 2;13:8995. doi: 10.1038/s41598-023-36174-5 (PMC10238405; doi:10.1038/s41598-023-36174-5)
Supplement: Supplementary file 1 — Supplementary Legends. [file 41598_2023_36174_MOESM1_ESM.doc]

Supplementary Materials for

**First lift-off and flight performance of a tailless flapping-wing aerial robot in high-altitude environments**

Shu Tsuchiya, Hikaru Aono, Keisuke Asai, Taku Nonomura, Yuta Ozawa, Masayuki Anyoji, Noriyasu Ando, Chang-kwon Kang, and Jeremy Pohly

Correspondence to: aono@shinshu-u.ac.jp

**This PDF file includes:**

Caption video S1 and S2

**Other Supplementary Materials for this manuscript include the following:**

Video S1 and S2

**Caption video S1**

Movie of a lift-off flight of the robo-hummingbird Shushu with SU Wing at lowest density condition. The air density is 0.360 kg/m^3^ corresponds to the flight altitude of 9,000 m. The flapping frequency is 10.9 Hz. The video was recorded at 30 frame per seconds (fps) using a smartphone (iPhone XR, Apple Inc.).

**Caption video S2**

Slow motion movie of a lift-off flight of the robo-hummingbird Shushu with SU Wing at lowest density condition. The air density is 0.360 kg/m^3^ corresponds to the flight altitude of 9,000 m. The flapping frequency is 10.9 Hz. The video was recorded at 2000 frame per seconds (fps) using highspeed camera (SA-X2, Photron).
